# High Intrinsic Aerobic Capacity Protects against Ethanol-Induced Hepatic Injury and Metabolic Dysfunction: Study Using High Capacity Runner Rat Model

**DOI:** 10.3390/biom5043295

**Published:** 2015-11-20

**Authors:** Nicholas Szary, R. Scott Rector, Grace M. Uptergrove, Suzanne E. Ridenhour, Shivendra D. Shukla, John P. Thyfault, Lauren G. Koch, Steven L. Britton, Jamal A. Ibdah

**Affiliations:** 1 Division of Gastroenterology and Hepatology, University of Missouri School of Medicine, Columbia, MO 65212, USA; E-Mails: nms.20007@gmail.com (N.S.); RectorS@health.missouri.edu (R.S.R.); meersg@health.missouri.edu (G.M.U.); ridenhoursuz@health.missouri.edu (S.E.R.); jthyfault@kumc.edu (J.P.T.); 2 Harry S. Truman Memorial Veterans Medical Center, 800 Hospital Drive, Columbia, MO 65201, USA; 3 Departments of Nutrition and Exercise Physiology, University of Missouri, Columbia, MO 65212, USA; 4 Departments of Medical Pharmacology and Physiology, University of Missouri, Columbia, MO 65212, USA; E-Mail: shuklasd@missouri.edu; 5 Departments of Anesthesiology, University of Michigan, Ann Arbor, MI 48109, USA; E-Mails: lgkoch@umich.edu (L.G.K.); brittons@umich.edu (S.L.B.); 6 Departments of Molecular & Integrative Physiology, University of Michigan, Ann Arbor, MI 48109, USA

**Keywords:** fatty liver disease, aerobic fitness, ethanol, mitochondrial function

## Abstract

Rats artificially selected over several generations for high intrinsic endurance/aerobic capacity resulting in high capacity runners (HCR) has been developed to study the links between high aerobic fitness and protection from metabolic diseases (Wisloff *et al.*, Science, 2005). We have previously shown that the HCR strain have elevated hepatic mitochondrial content and oxidative capacity. In this study, we tested if the elevated hepatic mitochondrial content in the HCR rat would provide “metabolic protection” from chronic ethanol-induced hepatic steatosis and injury. The Leiber-Decarli liquid diet with ethanol (7% *v*/*v*; HCR-E) and without (HCR-C) was given to HCR rats (*n* = 8 per group) from 14 to 20 weeks of age that were weight matched and pair-fed to assure isocaloric intake. Hepatic triglyceride (TG) content and macro- and microvesicular steatosis were significantly greater in HCR-E compared with HCR-C (*p* < 0.05). In addition, hepatic superoxide dismutase activity and glutathione levels were significantly (*p* < 0.05) reduced in the HCR-E rats. This hepatic phenotype also was associated with reduced total hepatic fatty acid oxidation (*p* = 0.03) and β-hydroxyacyl-CoA dehydrogenase activity (*p* = 0.01), and reductions in microsomal triglyceride transfer protein and apoB-100 protein content (*p* = 0.01) in HCR-E animals. However, despite these documented hepatic alterations, ethanol ingestion failed to induce significant hepatic liver injury, including no changes in hepatic inflammation, or serum alanine amino transferase (ALTs), free fatty acids (FFAs), triglycerides (TGs), insulin, or glucose. High intrinsic aerobic fitness did not reduce ethanol-induced hepatic steatosis, but protected against ethanol-induced hepatic injury and systemic metabolic dysfunction in a high aerobic capacity rat model.

## 1. Introduction

Alcoholic and non-alcoholic liver disease (NAFLD) are the two major causes of fatty liver disease. Alcoholic liver disease (ALD) is the third leading preventable cause of death in the United States accounting for 79,000 deaths annually [1] and steatosis secondary to obesity is the number one cause of elevated liver enzymes in the ambulatory population. NAFLD is a manifestation of the metabolic syndrome whose incidence is rising due to increased inactivity and availability of high calorie foods while excess ethanol intake is the driving force for ALD. ALD shares its histology with nonalcoholic counterpart where a spectrum of findings ranging from hepatic steatosis, alcoholic hepatitis and cirrhosis can be noted. Histological features of ethanol ingestion include hepatocyte ballooning, spotty necrosis, Mallory bodies and macrocytic steatosis, which are identical to nonalcoholic steatohepatitis (NASH) [2].

Excessive ethanol ingestion can cause mitochondrial dysfunction leading to disrupted function of the hepatocyte. Ethanol disrupts mitochondria by damaging mitochondrial DNA and causes effects on translation of proteins leading to inhibition of mitochondrial protein synthesis [3]. This leads to energy depravation for the hepatocyte and overproduction of reactive oxygen species leading to lipid accumulation and oxidative stress. Lipid accumulation, in turn, can lead to steatohepatitis and eventual cirrhosis.

Higher levels of aerobic fitness have been shown to be associated with reduced incidence of NAFLD in both animal models and human studies [4,5]. In addition, high aerobic fitness has been shown to be a good predictor of greater longevity while lower aerobic fitness is a predictor of early mortality [6]. Moreover, mitochondrial dysfunction has been linked to development of NAFLD and its progression to NASH [7]. Additionally, in humans the activity of mitochondrial respiratory chain complexes has been shown to be impaired in NASH [8].

A novel model in which rats are artificially selected over several generations for high and low intrinsic endurance/aerobic capacity resulting in high capacity (HCR) and low capacity (LCR) runners was developed to study the effects of high aerobic fitness and protection from metabolic disease [9]. We found that HCR rats are resistant to high fat diet induced obesity and insulin resistance by virtue of increased skeletal muscle oxidative capacity [10]. Additionally, we reported that the HCR strain has elevated hepatic mitochondrial content and oxidative capacity, and LCR rats have reduced hepatic mitochondrial oxidative capacity that increases the susceptibility to hepatic steatosis and liver injury [11]. HCR rats also show protection against high fat diet induced reactive oxygen species production in the kidney [12].

The paucity of literature regarding intrinsic aerobic capacity and ethanol intake has led us to investigate the relationship of the environmental stressor ethanol on high intrinsic aerobic capacity. The aim of this study was to determine if the elevated mitochondrial content and oxidative capacity in a rat bred for high aerobic capacity provided the ability to reduce the damaging effects from ethanol consumption on the liver. As ethanol is known to cause steatosis and inflammation of the liver, we hypothesize that HCR rats fed ethanol would be “metabolically” protected against hepatic steatosis and liver injury from ethanol.

## 2. Results

### 2.1. Animal Characteristics

There were no significant differences between HCR-C and HCR-E treated animals for body weight or serum glucose, insulin, FFAs (free fatty acids), or TGs (triglycerides) (Table 1). In addition, due to the pair feeding design, the food consumption also did not differ between groups. 

**Table 1:** Animal Characteristics. No significant differences were noted between the HCR-C (high capacity runner-control) and HCR-E (high capacity runner-ethanol) groups.

Parameters	HCR-C	HCR-E
Body Weight (grams)	311.1 ± 11.3	326.7 ± 11.6
Fat Pad Weight (grams)	21.5 ± 2.2	19.7 ± 1.8
Food consumption (g/week)	53 ± 2	53.1 ± 2.1
Serum Glucose (mg/mL)	195.8 ± 8.7	183.7 ± 9.2
Serum Insulin (ng/mL)	4.3 ± 0.5	4.9 ± 0.5
Serum Free Fatty Acids (umol/L)	285.4 ± 28.7	298.1 ± 27.9
Serum Triglycerides (mg/dL)	127.7 ± 3.6	123.3 ± 3.9

### 2.2. Hepatic Steatosis and Injury

**Figure 1:**
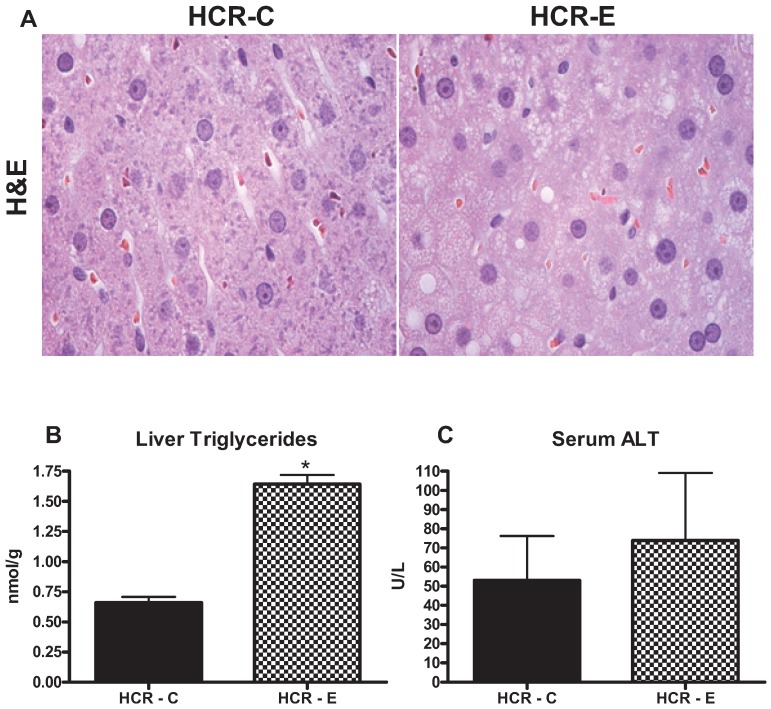
Effects of ethanol consumption on liver histology (**A**); liver TG accumulation (**B**); and serum ALT levels (**C**). (**A**) Representative H & E images; significant macro- and micro-vesicular lipid vacuolization were noted in HCR-E group (right panel). No inflammatory cells, hepatocyte ballooning or Mallory-Weiss bodies were noted; (**B**) Liver TG accumulation was significantly increased in the HCR-E animals; (**C**) Serum ALT levels were not different between the groups. Values (means ± SE) with * are significantly different (*p* < 0.05); *n* = 7–8/group.

Liver TG accumulation measured by lipid extraction was significantly greater in HCR-E group as compared to HCR-C group (*p* = 0.02) (Figure 1). Increased macro- and micro-vesicular steatosis also was noted in the HCR-E rats (Figure 1). No markers of injury such as ballooned hepatocytes, increased neutrophils or Mallory-Weiss bodies were noted (Figure 1). In addition, serum ALT levels did not differ significantly between HCR-E and HCR-C groups (Figure 1).

### 2.3. Fatty Acid Oxidation and Hepatic Mitochondrial Enzyme Activities

**Figure 2:**
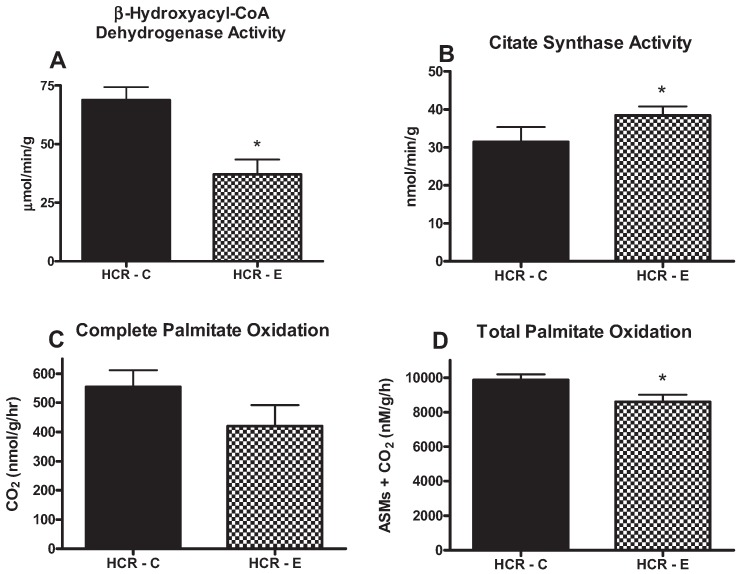
Effects of ethanol consumption on hepatic β-HAD activity (**A**); citrate synthase activity (**B**); complete (oxidation to CO_2_) hepatic palmitate oxidation (**C**); and total palmitate (CO_2_ + ASMs) oxidation (**D**). Values (means ± SE) with * are significantly different (*p* < 0.05); *n* = 7–8/group.

Ethanol consumption significantly reduced total palmitate oxidation (CO_2_ + ASMs; *p* = 0.03), but did not lower complete palmitate oxidation to CO_2_ (Figure 2). This may be explained by lower β-HAD activity, the rate-limiting step in fatty acid oxidation, which was also statistically lower in HCR-E animals (*p* = 0.01) (Figure 2). However, citrate synthase activity was significantly elevated in the HCR-E group (*p* < 0.05, Figure 2). Taken together, these data suggest reduced hepatic fatty mitochondrial function as a mechanism for hepatic steatosis in the HCR-E animals.

### 2.4. Markers of Oxidative Stress

**Figure 3:**
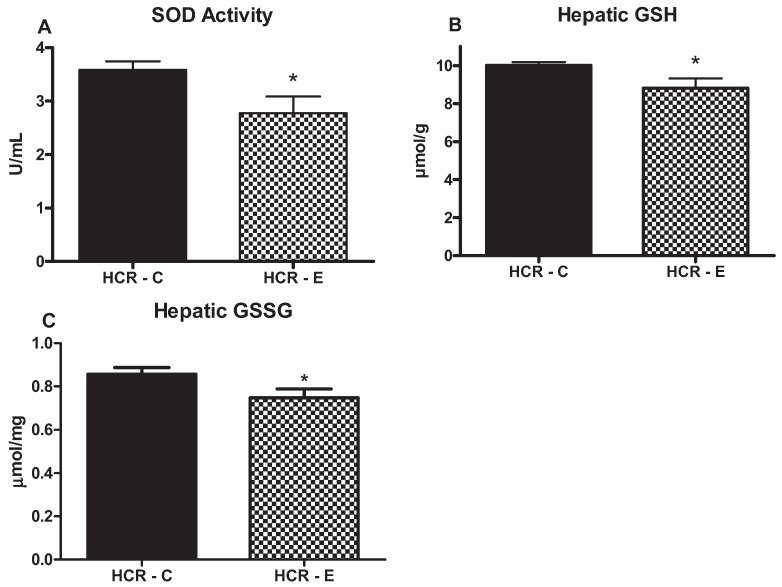
Effects of ethanol consumption on antioxidative markers superoxide dismutase (SOD) activity (**A**); reduced glutathione (GSH) (**B**); and oxidized glutathione (GSSG) (**C**). Values (means ± SE) with * are significantly different (*p* < 0.05); *n* = 7–8/group.

In order to examine the impact of chronic ethanol consumption on antioxidative parameters in the liver of the HCR rats, we assessed hepatic SOD activity, GSH and GSSG concentrations (Figure 3). Ethanol intake significantly reduced hepatic SOD activity and also concentrations of both reduced and oxidized glutathione compared with the HCR-C animals (*p* < 0.05) (Figure 3).

### 2.5. Markers of TG Export

**Figure 4:**
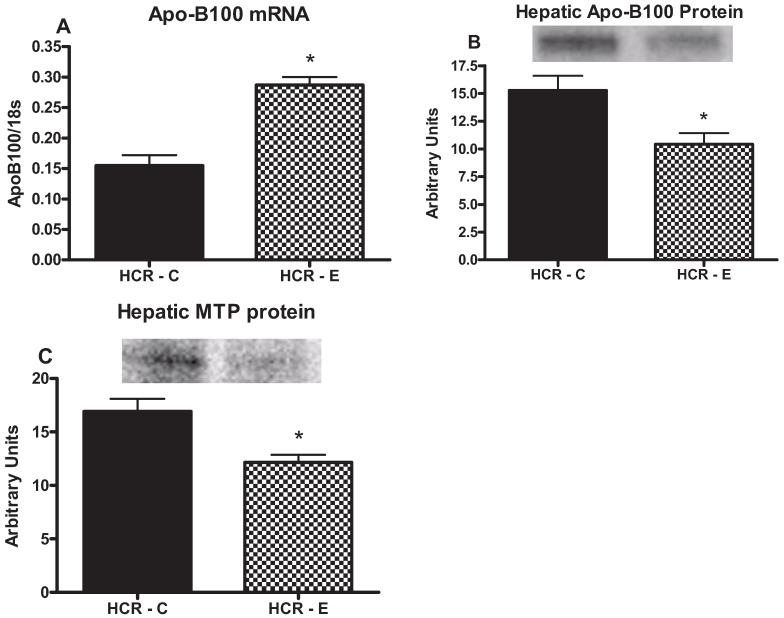
Effects of ethanol on markers of TG export apoB-100 mRNA (**A**); protein content of apoB-100 (**B**); and protein content of microsomal triglyceride transfer protein (MTP) (**C**). Values (means ± SE) with * are significantly different (*p* < 0.05); *n* = 7–8/group.

To assess the effects of chronic ethanol intake on markers of hepatic TG export, we examined the protein content of MTP, the rate limiting step in hepatic TG export from hepatocytes (Figure 4) and apoB-100 mRNA and protein content, which accepts TGs from MTP and the newly formed lipoprotein is then excreted from the hepatocyte. The addition of ethanol to HCR rats decreased both the hepatic apoB-100 and MTP protein content (*p* < 0.05, Figure 4). However, the level of apoB-100 mRNA was significantly increased (*p* < 0.05).

## 3. Discussion

In a previous study [11] we have shown that selection for low intrinsic aerobic capacity causes reduced hepatic mitochondrial content with increased susceptibility to both hepatic steatosis and liver injury. The aim of this study was to determine if high intrinsic aerobic capacity is protective against the damaging effects on the liver from ethanol consumption. Studies on Long Evans rats under similar experimental design have shown the strain to be highly susceptible to chronic (eight weeks) ethanol exposure which caused alterations in insulin resistance, increased oxidative stress, lipid peroxidation, and hepatocellular injury in liver [13,14]. Moreover, studies using Sprague-Dawley rat pair fed 5% ethanol over five weeks, showed significant increase in hepatic triglyceride concentration, alteration in mitochondrial number, as well as decrease in mitochondrial respiration [15]. No prior studies have examined the effects of ethanol administration on a polygenic rodent model with elevated hepatic mitochondrial content and increased lipid oxidative capacity. While rats bred for low aerobic capacity have been shown to have reduced mitochondrial oxidative capacity and susceptibility to hepatic steatosis under normal chow conditions, application of a stressor such as ethanol to that particular experimental model would not yield further insight into the mechanisms of hepatic steatosis. In contrast, testing if high aerobic capacity and associated higher hepatic mitochondrial content and function provide protection against ethanol induced steatosis and injury is a novel approach. Here we report that high intrinsic aerobic fitness did not protect against ethanol-induced hepatic steatosis, but it did protect against significant liver injury and systemic metabolic dysfunction. While chronic ethanol feeding has been shown to increase serum TG in human and rat studies [16], not all investigators have shown elevated TG levels under conditions of chronic ethanol consumption [7]. Here we report that serum TG, glucose and FFAs did not differ between control and ethanol treated groups. Previous studies on Long Evans and Sprague-Dawley rats documented changes in insulin as well as serum triglycerides as a result of chronic ethanol administration [14,15]. It is felt that HCR were protected from these negative alterations by virtue of their genetic makeup and elevated oxidative capacity. Intrinsic high aerobic capacity animals have been shown to have elevated basal hepatic oxidative capacity and be resistant to high fat diet obesity and insulin resistance [10]. These factors appear to be protective from alteration of serum lipid markers, insulin and glucose in our study.

High aerobic capacity and ethanol-induced hepatic steatosis: As noted in our results, ethanol induced hepatic steatosis in the ethanol fed HCR animals compared to the control group. The mechanisms of alcoholic steatosis include increased influx of fatty acids from ethanol-mediated lipolysis, impairment of fatty acid oxidation, and decreased export of fatty acids from the liver [17,18]. Each method contributes to increased levels of steatosis, which may in turn lead to further damage to the liver. In the current study, the addition of ethanol resulted in prominent micro- and macro-vesicular steatosis in the HCR rats. Dysregulated fatty acid oxidation and decreased export of fatty acids from the hepatocytes may play more prominent roles in hepatic steatosis development in the HCR-E animals. Ethanol increases hepatic steatosis through alcohol dehydrogenase-mediated ethanol metabolism, which generates the reduced form of nicotinamide adenine dinucleotide (NADH) and promotes steatosis by preventing/impairing fatty acid oxidation [17,19]. In our study, total fatty acid oxidation utilizing ^14^C-palmitate was lower in the HCR-E group. This finding is explained by lower β-HAD activity, the rate limiting step in fatty acid oxidation, which was also statistically lower in HCR-E animals. Previous work has shown that ethanol appears to depress a number of fully functional ribosomes thus decreasing mitochondrial protein synthesis [3,20]. Another feature of ethanol treated animals leading to hepatic steatosis was the level of proteins related to export of hepatic fatty acids. ApoB together with MTP form an assembly necessary for transfer of lipoproteins from hepatocytes [21]. Here we found that apo-B100 and MTP protein levels were significantly decreased in HCR**-**E animals as compared to control group, despite significant increases in apo-B100 mRNA in the HCR-E rats. While certain studies using HepG2 cells have shown transcription of MTP was reduced after the cells were exposed to ethanol [22,23], our study supports dysregulation at the level of the ribosome and impaired translation in HCR model. It is likely that the documented reduction in MTP levels led to reduced hepatic apo-B100 protein levels) due to rapid degradation of non-lipidated apoB-100. It is also likely that the documented reduction in apo-B100 protein level in hepatocytes has caused upregulation of apo-B100 transcriptional expression (due to feedback inhibition) resulting in increased apo-B100 mRNA levels.

High aerobic capacity protects against ethanol-induced hepatic injury: Our study documents that high intrinsic aerobic capacity protects against ethanol-induced hepatic injury such that the liver phenotype was limited to simple hepatic steatosis in the absence of other damaging effects. There were no signs of liver injury, such as increased neutrophils, ballooning of hepatocytes or Mallory-Weiss bodies, were noted. Moreover, serum levels of ALT, a marker of liver injury, were not significantly higher in the ethanol treated rats. Hepatic GSH and GSSG were significantly reduced in the HCR-E rats, suggesting a reduction in global amounts of glutathione available to the liver. These findings are in line with previous observations [24]. Additionally, SOD activity was significantly reduced in HCR-E animals. SOD removes excess superoxide from the electron transport chain thus preventing the ROS damage to the hepatic parenchyma [25]. Taken together, these findings suggest chronic ethanol consumption affects hepatic defense mechanisms without structural damage to hepatocytes in HCR animals.

Metabolism of ethanol takes place in the liver and mitochondrial dysfunction has been documented in alcoholic liver disease leading to oxidative stress and damage to hepatocytes [26]. Chronic ethanol administration also can lead to oxidative stress by increasing reactive oxygen species (ROS) through induction of cytochrome P450 system and through mitochondrial pathways by increasing the oxygen utilization in hepatocytes [27,28]. Overproduction of ROS can overwhelm hepatocellular defenses such as hepatic glutathione, thus leading to cellular damage and death. Oxidative stress can cause cellular apoptosis. One of the mechanisms proposed to explain alcohol-induced hepatocyte apoptosis is the release of cytochrome C in the cytosol where it promotes caspases activation. The role of alcohol in ROS production, the induction of the mitochondrial cell death pathway and the possible mechanisms involved have been recently reviewed [29].

The protective effect of high intrinsic aerobic capacity in the HCR rat model against ethanol induced hepatic injury is likely due to improved mitochondrial health, which coupled with the documented reduction in mitochondrial β-oxidation will lead to reduced ROS formation with downregulation of the antioxidants GSH and SOD. This in turn will lead to improved mitochondrial function and reduced release of cytochrome C leading to reduced apoptosis and enhanced anti-apoptotic process resulting in absence of inflammation (neutrophils, hepatocyte ballooning, Mallory-Weiss bodies) or fibrosis. Previously, we have shown that the HCR rats had a significantly lower percentage of apoptotic nuclei than their LCR counterparts supporting that high intrinsic aerobic capacity protects against apoptosis. Future studies are needed in this model to further assess oxidant/antioxidant status in ethanol treated mice including measurement of intracellular ROS levels and extra-mitochondrial oxidative capacity such as CYP2E1 activity. The concepts underlying the protective effect of high intrinsic aerobic capacity, specifically the role of mitochondria and the balance between oxidative stress and antioxidant response, and role of apoptosis have been noted in patients with nonalcoholic fatty liver disease as recently documented by Tarantino *et al.* [30,31].

In summary, high intrinsic aerobic fitness protects against ethanol-induced hepatic injury and systemic lipid and glucose alterations despite significant hepatic steatosis development.

## 4. Experimental Section

### 4.1. Animal Strains and Protocols

The animal protocols were approved by the Institutional Animal Care and Use Committees at the University of Missouri and the University of Michigan and the Subcommittee for Animal Safety at the Harry S. Truman Memorial VA Hospital. The development of LCR and HCR rats has previously been described [9,10]. Two-way artificial selective breeding was used to create low capacity runner (LCR) and high capacity runner (HCR) strains that were divergent for treadmill running capacity (run time to exhaustion on a graded exercise test). The N:NIH stock of rats served as the founder population [9]. In brief, the 13 lowest and 13 highest capacity rats of each sex were selected from the founder population and randomly paired for breeding. At each subsequent generation, within-family selection from 13 mating pairs was practiced for each line. The use of 13 families maintains a relatively low coefficient for inbreeding (<0.01/generation) and maximizes the retention of genetic variation. After the rats were phenotyped for running capacity, they were exposed to no further exercise training or testing and only underwent normal cage activity. For the current study, only the HCR animals were examined, because we have recently shown that the LCR rats are susceptible to the development of hepatic steatosis under normal low-fat chow dietary conditions [11].

The Lieber-Decarli liquid diet with and without ethanol (7% *v*/*v*) was given to HCR rats (14 to 20 weeks old) [26]. HCR rats were divided into ethanol (HCR-E) and control (HCR-C) groups (*n* = 8 per group) matched by their initial body weights and were pair fed to assure isocaloric intake. Treatment continued for six weeks in 12 h light/12 h dark cycle at 22 °C. Animals were fed daily and weighed weekly through the duration of the study. On the morning of animal sacrifice, the rats were injected with sodium pentobarbital (100 mg/kg), blood was collected from the aorta, animals were further exsanguinated and tissues were frozen at −80 °C, placed in RNAlater, or formalin-fixed.

### 4.2. Tissue Homogenization

Livers were quickly excised from anesthetized rats and either flash frozen in liquid nitrogen, placed in 10% formalin, or placed in ice-cold buffer (100 mM KCl, 40 mM Tris-HCl, 10 mM Tris-Base, 5 mM MgCl_2_·6H_2_O, 1 mM EDTA, and 1 mM ATP; pH 7.4). Fresh tissue hepatic fatty acid oxidation assays were performed as previously described by our group [32].

### 4.3. Fatty Acid Oxidation

Livers were quickly excised from anesthetized rats and placed in ice-cold isolation buffer (100 mM KCl, 40 mM Tris-HCl, 10 mM Tris-Base, 5 mM MgCl_2_·6H_2_O, 1 mM EDTA, and 1 mM ATP; pH 7.4). Palmitate oxidation was measured with radiolabeled [1-^14^C]palmitate (American Radiochemicals, St. Louis, MO, USA) in fresh liver homogenate preparations as previously reported [32]. Both ^14^CO_2_, representing complete fatty acid oxidation, and ^14^C labeled acid soluble metabolites (ASMs), representing incomplete fatty acid oxidation, were collected and counted as previously described [32].

### 4.4. Hepatic Lipid Content

Intrahepatic lipid content was obtained by weighing ~30 mg of liver and adding 1 mL of lipid extraction solution made from 1:2 (vol:vol) methanol:chloroform. The solution was homogenized for 30 s and left overnight at 4 °C with gentle shaking. One milliliter of 4 mM MgCl was added, vortexed, and centrifuged for 1 h at 1000× *g* at 4 °C. The aqueous phase was removed. The remaining organic phase was evaporated overnight, and 100 µL of 3:2 (vol:vol) butanol:triton-x114 was added. Lipid content was measured via a commercially available kit from Sigma (F6428) and TG concentrations were expressed as nmol/g wet weight as previously described [32].

### 4.5. Western Blotting

Western blot analyses were performed for the determination of the protein content of apo-B100 (Abcam, Cambridge, MA, USA) and microsomal triglyceride transfer protein (MTP; Santa Cruz Biotechnology Inc., Santa Cruz, CA, USA) using methods previously described [32,33]. In order to control for equal protein loading and transfer, the membranes were stained with 0.1% amido-black (Sigma, St. Louis, MO, USA) as previously described [32]. The total protein staining for each lane was quantified and these values were used to correct for any differences in protein loading or transfer of all band densities.

### 4.6. Serum Assays

Serum was collected from whole blood fraction by centrifuging whole blood for 10 min at 10,000 g. Serum was frozen at −80 °C and used for automated assay for AST and ALT by the Clinical Pathology Laboratory in the College of Veterinary Medicine at the University of Missouri using the Olympus AU400e Chemistry Immuno Analyzer (Olympus America, Inc., Center Valley, PA, USA). Serum insulin was measured by ELISA (Linco Research, St. Charles, Mo, USA). Serum glucose, TG, free fatty acids (FFA), and β-hydroxybutyrate concentrations were measured as previously described [32,34].

### 4.7. H & E and Staining of Liver Sections, SOD Activity and Glutathione Assays

To examine liver morphology, formalin-fixed paraffin embedded sections of liver were stained with hematoxylin and eosin (H & E) [13]. Liver superoxide dismutase (SOD) activity was determined by commercially available methods (Cayman Chemicals, Ann Arbor, MI, USA). GSH and GSSG concentrations were determined by a fluorometric method as previously described by our group [35].

### 4.8. Real-Time PCR

Apo-B100 mRNA expression was quantified by real-time PCR using the ABI 7000 Sequence Detection System and software. Liver samples were removed from RNA later and then pulverized in RLT buffer using the Qiagen^®^ TissueLyser system with subsequent RNA isolation from the RNeasy kit, with the optional DNaseI step (Qiagen, Valencia, CA, USA). Purity was ensured and concentration determined with a spectrophotometer (Nano Drop 1000, Thermo Scientific, Waltham, MA, USA). Reverse transcription was performed by combining RNA with the reverse transcription reaction mixture (Nuclease-Free Water, ImProm-II 5× Reaction Buffer, MgCl_2_, dNTP mix, and ImProm-II Reverse Transcriptase, (Promega, Madison, WI, USA) and cDNA was synthesized. The reaction mixture (Nuclease-Free Water, 18S, and apo-B100 primers and probe, both forward and reverse transcriptases, and TAQman Master Mix (ABI)) was loaded to a 96-well microplate, along with the cDNA sample (50 ng) and placed into the ABI 7000 Sequence Detection System for polymerization. After polymerization, results were quantified using the ddC_T_ method relative to 18S. Comparison of the differences in raw C_T_ values did not differ (*p* = 0.5) between groups, indicating that 18S mRNA was an appropriate normalizer.

### 4.9. Hepatic Mitchondrial Enzymes

Citrate synthase and beta-hydroxyacyl-CoA dehydrogenase (β-HAD) activities were determined in whole liver homogenate using spectrophotometric methods as previously described [32].

### 4.10. Statistics

Statistics were carried out by using software SPSS 15.0 (Chicago, IL, USA). Group differences were examined by independent samples *t*-test. Values are reported as means ± SE, and *p*-values of <0.05 were considered statistically significant.

## 5. Conclusions

High capacity runner rats were fed normal or ethanol containing liquid diet chronically for six weeks and various liver parameters were monitored. Ethanol ingestion did not induce significant liver injury including hepatic inflammation. High intrinsic aerobic fitness did not reduce ethanol induced hepatic steatosis, but protected against ethanol induced hepatic injury and systemic metabolic dysfunction in this rat model.

## Abbreviations

ALT: alanine aminotransferase; β-HAD: Beta-hydroxyacyl-CoA dehydrogenase; FFA: free fatty acids; MTP: microsomal triglyceride transfer protein.
